# DIALAPP: a prospective validation of a new diagnostic algorithm for acute appendicitis

**DOI:** 10.1007/s00423-020-02022-7

**Published:** 2020-11-19

**Authors:** Patrizia Malkomes, Franziska Edmaier, Juliane Liese, Alexander Reinisch-Liese, Hanan El Youzouri, Teresa Schreckenbach, Andreas M. Bucher, Wolf Otto Bechstein, Andreas A. Schnitzbauer

**Affiliations:** 1 Department of General, Visceral and Transplant Surgery, University Hospital Frankfurt, Goethe University, Theodor-Stern-Kai 7, 60590 Frankfurt am Main, Germany; 2 Department of General, Abdominal, Thoracic and Transplant Surgery, University Hospital of Giessen, Giessen, Germany; 3 Department of General, Visceral and Oncological Surgery, Wetzlar Clinic, Wetzlar, Germany; 4 Department of Radiology, Institute for Diagnostic and Interventional Radiology, Goethe University Hospital Frankfurt, Frankfurt am Main, Germany

**Keywords:** Acute appendicitis, Diagnostic algorithm, Risk-stratification, Negative appendectomy rate, Clinical trial

## Abstract

**Purpose:**

The management of patients with suspected appendicitis remains a challenge in daily clinical practice, and the optimal management algorithm is still being debated. Negative appendectomy rates (NAR) continue to range between 10 and 15%. This prospective study evaluated the accuracy of a diagnostic pathway in acute appendicitis using clinical risk stratification (Alvarado score), routine ultrasonography, gynecology consult for females, and selected CT after clinical reassessment.

**Methods:**

Patients presenting with suspected appendicitis between November 2015 and September 2017 from age 18 years and above were included. Decision-making followed a clear management pathway. Patients were followed up for 6 months after discharge. The hypothesis was that the algorithm can reduce the NAR to a value of under 10%.

**Results:**

A total of 183 patients were included. In 65 of 69 appendectomies, acute appendicitis was confirmed by histopathology, corresponding to a NAR of 5.8%. Notably, all 4 NAR appendectomies had other pathologies of the appendix. The perforation rate was 24.6%. Only 36 patients (19.7%) received a CT scan. The follow-up rate after 30 days achieved 69%, including no patients with missed appendicitis. The sensitivity and specificity of the diagnostic pathway was 100% and 96.6%, respectively. The potential saving in costs can be as much as 19.8 million €/100,000 cases presenting with the suspicion of appendicitis.

**Conclusion:**

The risk-stratified diagnostic algorithm yields a high diagnostic accuracy for patients with suspicion of appendicitis. Its implementation can safely reduce the NAR, simultaneously minimizing the use of CT scans and optimizing healthcare-related costs in the treatment of acute appendicitis.

**Trial registration:**

ClinicalTrials.gov Identifier: NCT02627781 (December 2015)

## Introduction

With an incidence of 120 per 100,000 inhabitants, appendicitis is one of the most common causes of acute abdomen in Germany. The lifetime prevalence for appendicitis is approximately 7%, making appendectomy one of the most frequently performed operations with 110,000 procedures per year [1, 2]. However, the diagnosis of acute appendicitis (AA) continues to be a challenge even for experienced surgeons resulting in a mean negative appendectomy rate (NAR) of 10 to 15% [3–5].

As negative appendectomies are associated with the potential of substantial morbidity and increased healthcare-related costs, they should be reduced to a minimum [6]. Several scoring systems and modern imaging modalities have been implemented in an attempt to increase the diagnostic accuracy as they have shown to reduce NAR. Studies from the USA could reduce the NAR to 3.2% by performing a computed tomography (CT) scan in each patient presenting with right-sided lower abdominal quadrant pain [7]. Nevertheless, non-selective imaging shows limited accuracy in subgroups with a low or high clinical probability of appendicitis, suggesting that improvements in diagnostic outcomes are dependent on the appropriate selective application of CT, and the integration of clinical findings with CT results [8, 9]. Moreover, several authors have criticized the widespread use of CT due to the negative impact of ionizing radiation exposure in this generally young patient population, raising concerns about cancer risk [10, 11].

In 1986, Alvarado et al. [12] devised a simple diagnostic score for the diagnosis of acute appendicitis based on the probability of clinical signs (tenderness in right lower quadrant, rebound pain, and elevated temperature > 37.5 °C), symptoms (pain migration to right lower quadrant, anorexia, and nausea/vomiting), and laboratory testing (leukocytosis and neutrophils > 75%) for predicting appendicitis (Table 1). In subsequent studies, the Alvarado score has been validated showing a good sensitivity but a considerably low specificity of only 81% [13].

**Table 1:** Alvarado scoring system for acute appendicitis

Characteristics	Score
Symptoms	
Pain migration to right lower quadrant	1
Anorexia	1
Nausea/vomiting	1
Clinical signs	
Tenderness in right lower quadrant	2
Rebound pain	1
Elevated temperature > 37.5 °C	1
Laboratory	
Leukocytosis	2
Neutrophils > 75%	1
Total	10

**Fig. 1:**
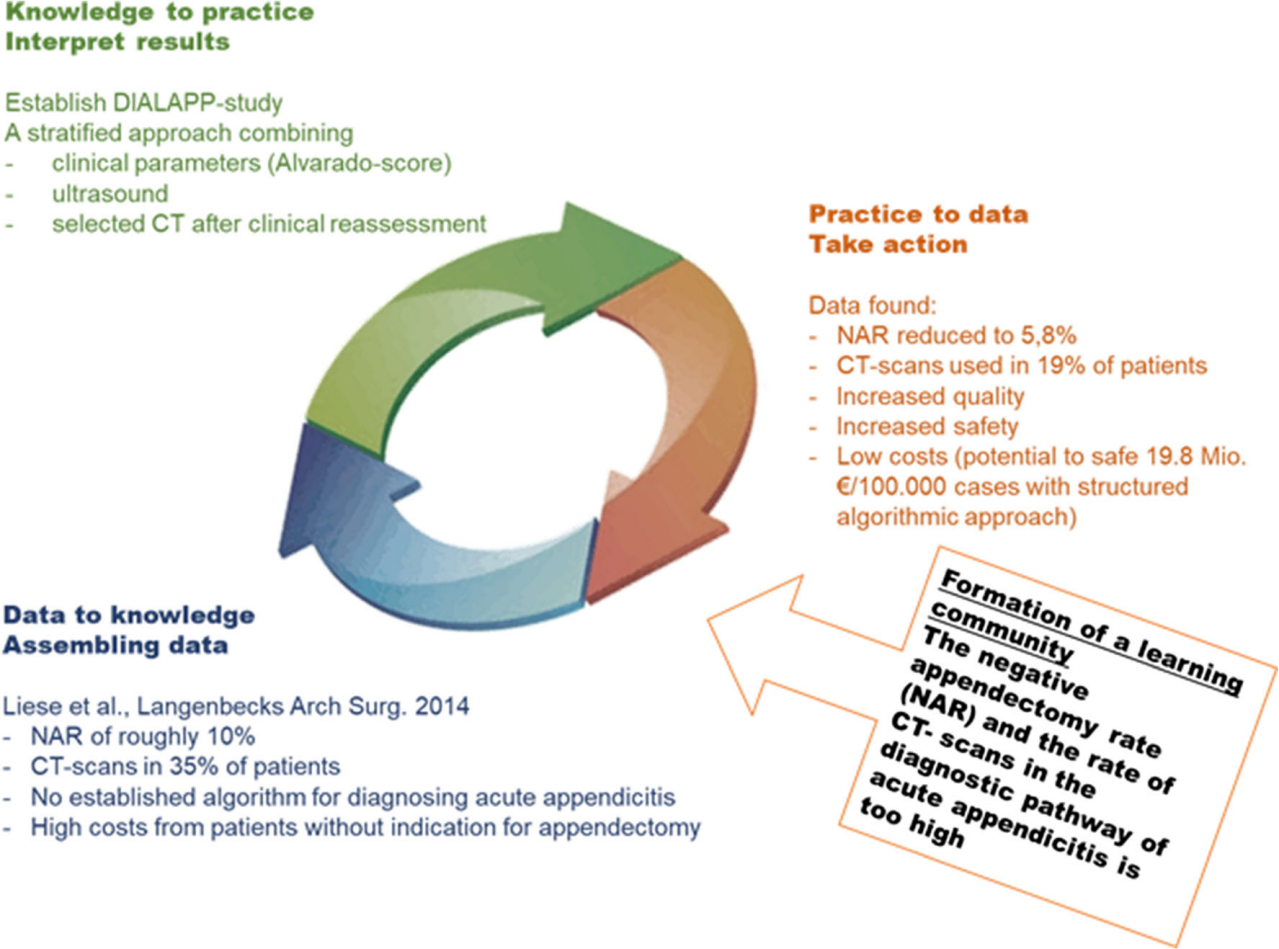
Learning circle of the DIALAPP study. Starting with the formation of a learning community in order to minimize the rate of negative appendectomies and CT scans in the diagnosis of acute appendicitis. After the analysis of our institutional retrospective data of Liese et al. (data to knowledge), a diagnostic pathway was implemented and prospectively evaluated as part of the DIALAPP study (knowledge to practice). As a result, we found a reduction in the NAR and CT rate (practice to data) (NAR, negative appendectomy rate)

Several studies have determined the diagnostic accuracy of individual diagnostic procedures and their combinations in the diagnosis of AA [14–17]. However, there is little evidence for the application of diagnostic pathways, and none is in routine clinical use. In a study at our institution by Liese et al. [18], data from 367 patients with suspected appendicitis were retrospectively analyzed, and it was found that the overall NAR was 10.1% with a simultaneous CT rate of 35%. Patients who had received a CT scan showed a lower NAR of 5.7% in comparison with those who had not undergone imaging with a frequency of negative appendectomies of 11.7%. Thereby, CT imaging helped to reduce total hospital expenses. However only 32% of patients undergoing CT benefitted from this diagnostic modality, and thus, a relevant proportion of CT scans could have been avoided [18]. Based on these retrospective findings, a clinical diagnostic algorithm was developed and implemented. The DIALAPP study (evaluation of DIagnostic ALgorithm for suspected acute APPendicitis) is a prospective observational unicentric study to evaluate the implementation and impact of the newly established diagnostic pathway in patients with suspicion of acute appendicitis. The hypothesis was that the pathway reduces the NAR to less than 10%, while simultaneously reducing the use of CT imaging. The increased quality and safety should lead to a reduction in costs and create a real value for healthcare (Fig. 1).

## Methods

A clinical diagnostic algorithm was implemented at the Department for General and Visceral Surgery of the Goethe University Hospital Frankfurt starting in November 2015, which was applied to all patients over 18 years presenting with the symptoms of an acute appendicitis.

Written informed consent was obtained from all patients prior to inclusion in the study in accordance with the Declaration of Helsinki and local laws and regulations. The study was approved by the institutional ethics review board (IRB-Number: 268/15) and registered at clinicaltrials.gov (NCT02627781).

All consecutively included patients were evaluated by a resident or a consultant of the surgical department. Patients with abdominal pain due to trauma, patients under 18 years, and those who had undergone additional radiological examinations (CT or magnetic resonance imaging (MRI)) prior to surgical consultation were excluded.

### Clinical diagnostic pathway and data collection

All patients followed a structured diagnostic algorithm including all elements of the Alvarado score (Table 1), besides other parameters. First, a clinical examination, a biochemical and hematological blood analysis, urine analysis, as well as an ultrasound by a resident and/or consultant surgeon were performed. Additionally, all female patients of childbearing age received a gynecological consultation. Thereafter, the Alvarado score was calculated for each individual patient. All clinical parameters and the management strategy were recorded on a study sheet. Patients with signs of peritonitis, positive ultrasound, or an Alvarado score of > 8 were advised to undergo appendectomy. Patients with an Alvarado score of < 5 and negative ultrasound were discharged to outpatient follow-up. Those with an Alvarado score between 5 and 8 were admitted to observation and clinical re-evaluation. These patients only received symptomatic therapy (infusion therapy, laxative measures, analgesia) but no antibiotic treatment. The clinical course was assessed within 24 h after admission by a board-certified or consultant surgeon. Patients developing clinically suspected AA were advised to undergo appendectomy. The improvement of complaints resulted in discharge and outpatient follow-up. All patients with persistent abdominal complaints during the observation period received a CT or MRI (pregnant patients). Patients with a positive CT scan were indicated to undergo appendectomy. Those with a negative CT were discharged. Patients with alternative diagnoses on ultrasound, CT, or at the gynecological consultation received appropriate non-study specific treatment. During the application of the pathway between November 2015 and August 2017, patients were consented for prospective data collection. A telephone follow-up of all patients was undertaken after 30 days and 6 months.

The definitive diagnosis of appendicitis was determined by the final pathological report of the resected specimens. Histopathological criteria for acute appendicitis were granulocytic infiltration or ulceration of the lumen or all wall layers, as well as the fatty tissue. Another criterion was the presence of an intramural abscess or necrosis.

The appendicitis was classified as perforated based on intraoperative findings or pathological examination of the resected organs.

A negative appendectomy was characterized by the removal of an appendix without signs of acute inflammation in the pathological examination.

All CT scans covered the entire abdomen and pelvis, ranging from above the diaphragm to the inguinal region. Acquisition protocols were either a standard-dose abdominal and pelvic CT with primary intravenously contrast or a non-contrasted low-dose CT. All CT scans were acquired on a third-generation dual-source CT or a 64-slice multi-detector CT (Somatom Force, Somatom Definition AS+, Siemens). All scans were interpreted by two independent radiology readers and signed by senior radiology residents. CT studies were classified in a binary fashion as positive or negative for the presence of appendicitis. Patients not treated according to the algorithm and all cases with incomplete study sheets were excluded from the analysis.

The primary endpoint of the study was the NAR. Secondary endpoints included accuracy of the diagnostic tools, perforation rate, duration of diagnosis, complication rate, and length of hospital stay.

### Economic modeling

Liese et al. [18] described the cost of appendectomies in relation to undergoing surgery with or without CT scans or avoiding surgery utilizing a CT scan. We transferred their model to our setting and calculated the costs with the same pricing to create comparability. Pricing for the treatment of appendicitis indeed did not change in Germany during the mentioned time. The costs were calculated from the ratio of patients undergoing surgery with or without CT imaging and patients having no surgery due to the utilization of a CT. Moreover, we included a model with all patients (additional those that received no CT scan and no surgery due to the algorithm) and extrapolated the numbers hypothetically obtained from the data in the publication by Liese et al. [18] to receive a narrowing estimation of the overall potential in saving expenses and costs for 100,000 cases of suspected AA.

### Data analysis

Statistical analysis was performed using BiAS (Version 11.08) and Microsoft Excel 200x (Microsoft Corporation). Values are reported as median with range or proportions. Categorical variables were compared by means of the chi-squared test or Fisher’s exact test and independent variables using the Mann-Whitney *U*-test or the Shapiro-Wilk test as appropriate. A *p* value of < 0.05 was considered to be statistically significant.

## Results

### Patient selection, decision tree, and drop-out rate

**Fig. 2:**
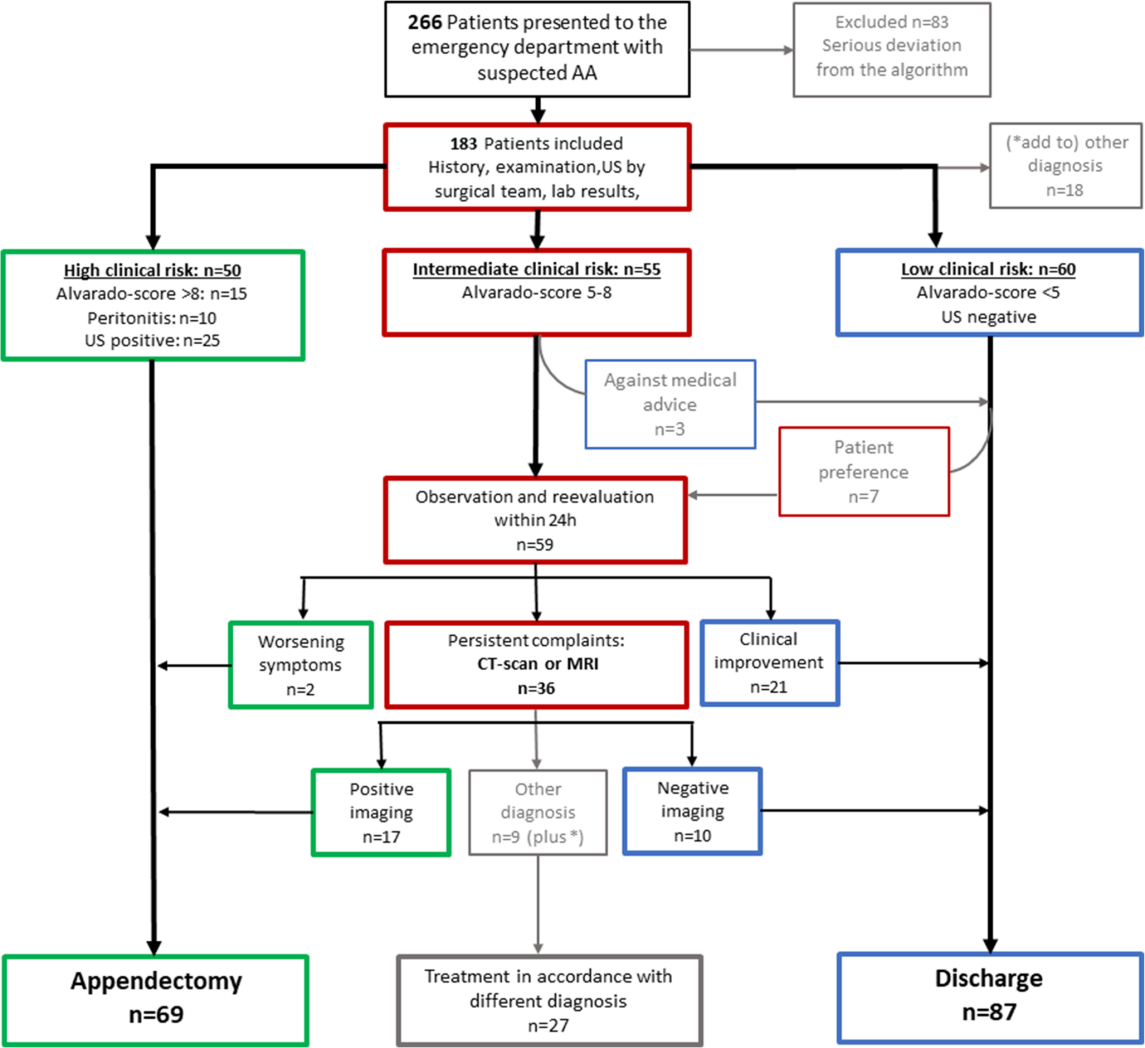
Flowchart and management course of study cohort. Implementation of the proposed diagnostic algorithm in 183 patients with suspected appendicitis (AA, acute appendicitis; US, ultrasound)

A total of 266 patients were evaluated with suspected acute appendicitis during the study period. Of these, 83 patients were excluded due to severe protocol violation. Most common reasons for exclusion were missing gynecological consultation (*n* = 37), missing ultrasound (*n* = 12), missing differential blood count (*n* = 10) or discharge with an Alvarado score of > 4 (*n* = 7), observation with Alvarado score of < 4 (*n* = 5), and a CT imaging in patients with an Alvarado score of < 5 (*n* = 6). In total, 183 patients were included in the study and final analysis. Figure 2 shows a flowchart of the implementation of the standardized diagnostic pathway. Sixty-nine patients (37.7%) underwent appendectomy based on clinical features (*n* = 27; 39.1%), the evidence of appendicitis on ultrasound (*n* = 25; 36.2%), or on a CT scan (*n* = 17; 24.6%). In 55 patients, the Alvarado score (5–8) and ultrasound were inconclusive. These patients were clinically observed. Of those, 21 patients recovered without operation and were discharged. Thirty-six patients with persisting abdominal complaints underwent additive CT imaging. Hereof, 17 CT scans showed signs of appendicitis, and all underwent surgery, and the diagnosis of appendicitis was confirmed in the pathology workup. In 19 patients, the CT was negative for appendicitis, and 9 CT scans determined a different diagnosis. In total, 87 patients (47.5%) could be discharged with resolved abdominal problems, of whom only 10 (11.4% of the discharged and only 19.7% of all study patients) received a CT. In 27 patients, a different diagnosis than appendicitis could be determined following the diagnostic pathway.

### Patient characteristics with a proven appendicitis and with no proof for appendicitis

In total, 183 were analyzed as defined per protocol. Patients with appendicitis were significantly older than patients without appendicitis (32, range 18–71 vs. 28, range 18–71; *p* = 0.0004). Significantly more female patients did not have appendicitis (no 69 vs. yes 22), whereas more male patients were diagnosed with appendicitis (no 49 vs. yes 43) (*p* = 0.0002). The clinical parameters of white blood cell count, C-reactive protein and the median Alvarado score were significantly higher for all items in patients with AA (*p* < 0.001). The hospital stay was significantly longer in patients with appendicitis (4 days vs. 3 days; *p* < 0.001). Data are displayed in Table 2.

**Table 2:** Demographic and clinical characteristics of patients with proven appendicitis in comparison with patients without appendicitis

	All (*n* = 183)	Appendicitis (*n* = 65)	No appendicitis (*n* = 118)	*p* value
Age (years)				0.0004
Median	30	32	28	
Range	18–71	18–61	18–71	
Sex				0.0002
Female	91 (49.7)	22 (33.8)	69 (58.5)	
Male	92 (50.3)	43 (66.2)	49 (41.5)	
BMI (kg/m^2^)				0.0018
Median	24.1	24.7	22.6	
Range	16.8–41.2	17.4–41.2	16.8–30.1	
Leukocytes (4.2–10/nl)				< 0.000001
Median	10.4	14.48	8.89	
Range	2.83–24.99	2.83–24.99	2.92–23.01	
C-reactive protein (< 0.5 mg/dl)				0.000011
Median	0.5	1.73	0.28	
Range	0.01–23.66	0.02–23.66	0.01–20.57	
Alvarado score				< 0.0000001
Median	5	7	4	
Range	0–10	4–10	0–8	
CT scan performed	36 (19.7)	17 (26.2)	19 (16.1)	0.1
Negative appendectomy	--	--	4 (5.8)	--
Length of hospital stay (days)				0.0001
Median	3	4	3	
Range	1–12	3–12	1–9	

Values are number of cases (%), unless otherwise indicated. For analysis of categorical variables, chi-squared test was used. Fisher’s exact test was used, if appropriate. Independent variables were compared with the Mann-Whitney *U* test or the Shapiro-Wilk test as appropriate

*BMI* body mass index

### Performance values of the Alvarado score, ultrasound, and the diagnostic algorithm

**Fig. 3:**
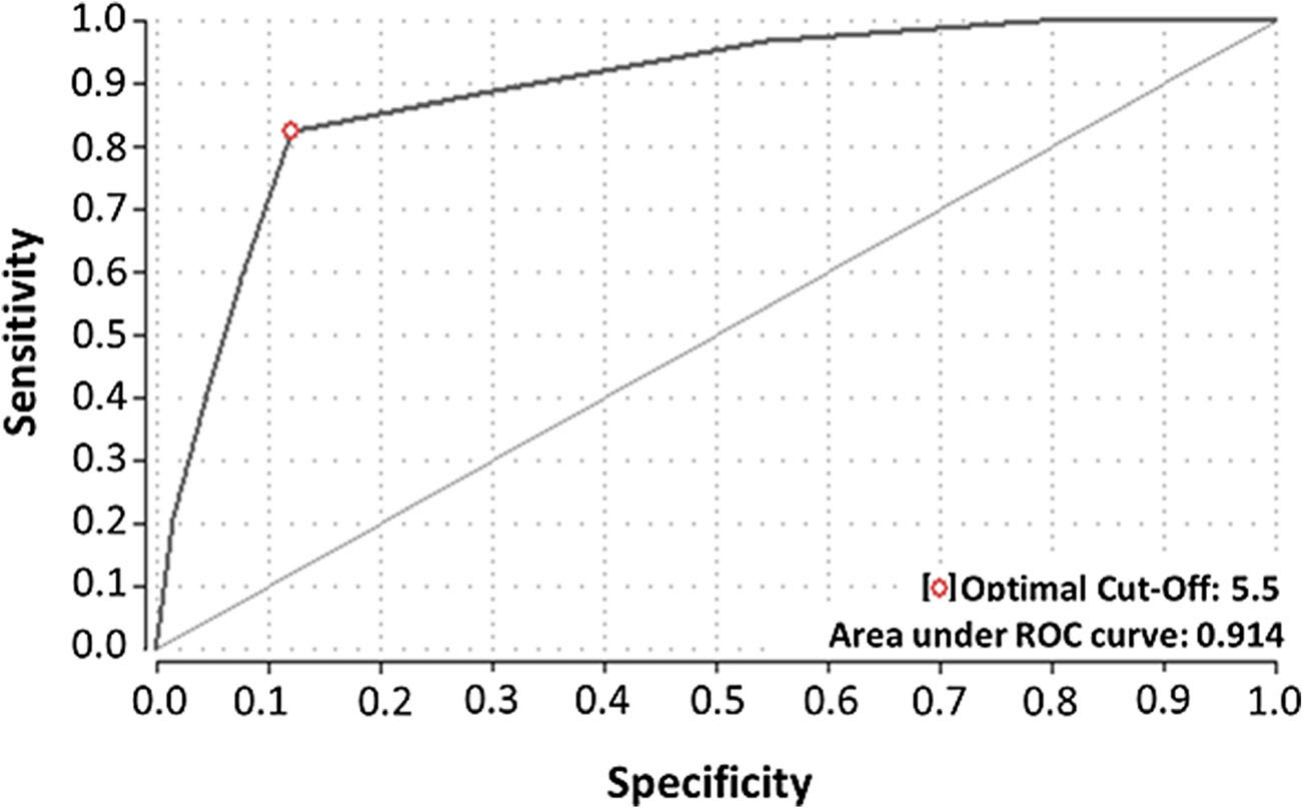
ROC curve. ROC (receiver operating characteristic) curve of sensitivity and specificity of the Alvarado score for diagnosis of acute appendicitis

The ROC (receiver operating characteristic) analysis identified a cutoff Alvarado score of 5.5 to identify patients at a high risk for AA and yielded a sensitivity of 92.4% and a specificity of 59.1%. The area under the ROC curve showed good diagnostic capacity (0.914; Fig. 3). Setting the cutoff value of the Alvarado score to > 7 as recommended in the diagnostic algorithm, the sensitivity and specificity are 73.9% and 88.1%, respectively. The ultrasound displayed a moderate sensitivity of 58.2% and high specificity of 97.3%. In contrast, CT was equally high concerning sensitivity and specificity with 100% respectively. In summary, the whole diagnostic algorithm, consisting specifically of (1) Alvarado score, (2) gynecological consultation for females, (3) ultrasound, and (4) selective CT imaging showed a sensitivity of 100% and a specificity of 96.6% for the detection of AA. Overall performance values for the Alvarado score, ultrasound, CT, and the entire diagnostic algorithm are shown in Table 3.

**Table 3:** Overall performance values for Alvarado score, ultrasound, CT, and diagnostic algorithm

Measurement	Alvarado-score > 7 (*n* = 183)		Ultrasonography (*n* = 181)		CT scan (*n* = 36)		Diagnostic algorithm (*n* = 183)	
	%	95% CI	%	95% CI	%	95% CI	%	95% CI
Sensitivity	73.9	61–84	58.2	35–62	100	81–100	100	95–100
Specificity	88.1	81–93	97.3	92–99	100	82–100	96.6	92–99
PPV	77.4	65–87	90.0	73–98	100	81–100	94.2	86–98
NPV	85.9	78–92	78.9	71–85	100	82–100	100	97–100
Accuracy	82.3	76–87	80.9	74–87	100	92–100	97.8	95–99

*PPV* positive predictive value, *NPV* negative predictive value

In 65 of 69 patients undergoing appendectomy, an acute appendicitis could be confirmed in the histopathological examination of the resected organs. This accounts for a NAR of 5.8%, and female patients had a NAR of 4.4%, and male patients, of 6.5%. In 17 patients (24.6%), a perforated appendicitis was found. Table 4 shows the characteristics of patients with acute appendicitis in comparison with patients with perforated appendicitis. Patients with a perforated appendicitis were older and more often male. There was no significant difference between both groups concerning laboratory parameters, the Alvarado score, or the rate of CT-scans. The time from admission to operation was longer in patients with uncomplicated appendicitis (424 min vs. 324 min), without reaching statistical significance. Fifteen patients (25.4%) who were operated within 12 h after admission showed a perforation in comparison with 2 patients (20.0%) being operated after 12 h, indicating that the time from admission to operation had no significant influence on perforation rate. Nevertheless, performing a preoperative CT scan resulted in a significantly delayed operation (599 min in patients with preoperative CT vs. 340 min in patients without CT; *p* = 0.005).

**Table 4:** Comparison of patients with uncomplicated and perforated appendicitis

	All (*n* = 65)	No perforation (*n* = 48)	Perforation (*n* = 17)	*p* value
Age (years)				0.05
Median	32	32	34	
Range	18–61	18–61	22–59	
Sex				0.46
Female	22 (33.8)	15 (31.3)	7 (41.1)	
Male	43 (66.2)	33 (68.7)	10 (58.9)	
Leukocytes (4.2–10/nl)				0.66
Median	14.48	14.83	13.79	
Range	2.83–24.99	2.83–24.37	3.2–24.99	
C-reactive protein (< 0.5 mg/dl)				0.08
Median	1.73	1.15	3.5	
Range	0.02–23.66	0.02–23.66	0.06–15.4	
Alvarado score				0.92
Median	7	7	7	
Range	4–10	4–10	6–10	
CT scan performed	17 (26.2)	12 (25.0)	5 (29.4)	0.41
Time to operation (min)				0.54
Median	404	424	357	
Range	141–5660	141–1559	146–5660	
Length of hospital stay (days)				0.004
Median	4	3	4	
Range	3–12	3–7	3–12	
Postoperative complications	10 (15.4)	7 (14.6)	3 (17.6)	0.55
Dindo-Clavien I-II	8	6	2	
Dindo-Clavien ÍII	2	1	1	
Dindo-Clavien IV-V	0	0	0	

Values are number of cases (%), unless otherwise indicated. For analysis of categorical variables, chi-squared test was used. Fisher’s exact test was used, if appropriate. Independent variables were compared with the Mann-Whitney *U* test or the Shapiro-Wilk test as appropriate

*BMI* body mass index

Patients with perforated appendicitis showed a longer hospital stay in comparison with patients with uncomplicated appendicitis. However, in our patient cohort, we could not detect a higher morbidity due to perforated appendicitis (Table 4). Sixty-eight patients underwent laparoscopic appendectomy. In one case, a conversion to open surgery was necessary (1.5%). One pregnant patient underwent open appendectomy simultaneously with cesarean section. Negative explorations did not occur in our cohort.

The histopathological examination of all of the four patients with negative appendectomy showed an abnormal result, including neurogenic appendicopathy (*n* = 2), adenoma of appendix (*n* = 1), and transmural fibrosis of appendix (*n* = 1).

Eleven patients had postoperative complications, of whom 2 had to undergo re-do surgery. One of these patients initially received a negative appendectomy and had to be reoperated due to bowel obstruction. One patient developed an intraabdominal collection and was treated with percutaneous drainage. Four patients developed surgical site infection (Table 4).

### Midterm follow-up at 30 days and 6 months

A total of 68.9% and 65.6% of all patients could be reached by telephone follow-up after 30 days and 6 months, respectively. No missed appendicitis occurred among the non-operated patients during the follow-up. Five patients after appendectomy were readmitted due to postoperative complications, of whom one patient had to undergo re-do surgery due to intestinal obstruction. Other reasons for hospital readmission were an intraabdominal abscess, a gastrointestinal motility disorder, and a myocardial infarction.

### Reduction of the costs by utilizing the new DIALAPP algorithm

The patients were categorized into 4 groups of costs: 1: no CT scan/surgery (1317 €), 2: CT scan/surgery (1434 €), 3: CT scan/no surgery (675 €), and 4: no CT scan/no surgery (558 € in hospital, 250 € in ambulatory setting). The groups were distributed by 52/59% for group 1, 17/19% for group 2, and 19/22% for group 3, resulting in 125.9 million € per 100,000 cases in the Liese et al. publication (historical patient cohort of our institution) and 119.2 million € for the present patient cohort. This accounted for a saving of 6.7 million € per 100,000 suspicious cases for AA. The extrapolated model included group 4 which was missing in the publication by Liese et al. We used the assumption that the reduction in NAR by 5% predominantly was observed in the no CT/surgery group and extrapolated the corresponding numbers accordingly: 34/28.4% for group 1, 12.5/9.2% for group 2, 5.9/10.3% for group 3, and 10 (ambulatory) + 37.6 (in hospital)/10 + 41.9% for group 4, resulting in 93.4 million € vs. 73.6 million € and a theoretic saving of 19.8 million € per 100,000 cases when applying all 4 categories.

## Discussion

The prospective diagnostic pathway of diagnosing acute appendicitis combining a clinical risk scoring (Alvarado score), routine ultrasound, highly selective use of CT imaging, and clinical re-evaluation showed a high accuracy with a sensitivity and specificity of 100% and 96.6%, respectively. The implementation of the algorithm resulted in substantial improvements in surgical diagnostic accuracy when compared with our previous institutional data [18]. As shown in Table 5, the implementation led to a reduction in the rate of unnecessary operations (the NAR was reduced to 5.8%) and an optimization of performed CT imaging (only 19% of all patients) and accelerated the access to adequate surgical treatment (mean time to operation, reduction from 684.2 to 555.6 min), which was considerably better than the former published experience by our group [18]. In the presented patient cohort, only four negative appendectomies occurred. Ultimately, in all four cases, histological results showed a pathological finding of the appendix, including neurogenic appendicopathy. Patients who had low clinical suspicion for appendicitis and negative ultrasound were safely discharged from the hospital, which was confirmed during the follow-up period, as we did not detect a missed appendicitis. Thus, the most important finding of the prospective single-armed interventional trial was the improvement in three surrogates of treatment quality for acute appendicitis derived from a first learning circle and retrospective analysis in a learning health system (Fig. 1) [18].

**Table 5:** Patient demographics and clinical outcome among patients in the pre-group in comparison to the pathway group

	Pre-group (*n* = 367)	Pathway group (*n* = 183)
Age (years)		
Mean	37.69	31.8
Range	15–91	18–71
Female gender (%)	158 (43.1)	91 (49.7)
Body mass index (kg/m^2^)		
Mean	24.9	24.4
Range	16.3–63.8	16.8–41.2
Preoperative CT (%)	129/367 (35.1)	36/183 (19.7)
Negative appendectomy rate (%)	33/326 (10.1)	4/69 (5.8)
Male	10/187 (5.3%)	3/46 (6.5%)
Female	23/139 (16.5%)	1/23 (4.4%)
Appendiceal perforation rate (%)	48/293 (16.4)	17/65 (26.1)
Time to operation (min)		
Mean	684.2	555.6
Range	52–100,004	141–5660

Pre-group, historical, retrospective patient cohort of our institution before application of the diagnostic pathway (published by Liese et al. [18]); pathway group, patient cohort during application of the diagnostic pathway

A number of studies describing a pathway for diagnosing patients with suspected appendicitis have been published. Only few of them have prospectively evaluated their recommended algorithm [14, 16, 17, 19]. In these studies, the implementation of a diagnostic pathway also resulted in a low rate of negative appendectomies of less than 10%. Nevertheless, the low NAR could only be achieved by a widespread use of CT scans in patients presenting with suspected appendicitis [16]. For example, Antevil [17] and co-authors observed a NAR of 4% with a simultaneous frequency of CT of 87%, probably due to a lack of clinical risk stratification before imaging. Similarly, other studies have demonstrated a decrease of NAR by an increased use of preoperative CT imaging [20–22]. Taking into account the radiation exposure, contrast-related complications, and resource consumption related to CT, some studies have implemented diagnostic pathways including clinical risk stratification before imaging in an attempt to reduce the rate of CT scans. Thus, in the STRAPPSCORE study [14], the introduction of a clinical score-based risk stratification algorithm in patients with suspicion of appendicitis resulted in less imaging and fewer admissions. But on the other hand, the algorithm did not show an effect on the proportion of negative appendectomies. Relating the number of negative appendectomies to all operated patients, the NAR was about 11%, with a maximum of 17.9% in patients with low risk for appendicitis.

Comparable with the current study, Toorenvliet and colleagues [19] demonstrated a NAR of 3.3% with a minimal use of CT in only 17.9% of all patients by the implementation of a diagnostic pathway including risk stratification and regular ultrasound. However, it must be noted that in this study, the patient risk stratification was undertaken by a consultant and not according to an objective clinical scoring system. In addition, both children and adults were investigated in the study, which certainly affects the data due to the restrained use of CT imaging in children.

The results of additionally required and indicated imaging in accordance with the algorithm in the DIALAPP study were excellent. Most strikingly, CT imaging had a 100% accuracy in the diagnosis of appendicitis (no false positive and no false negative results). This corresponds to the recent literature [9, 23, 24]. Here, the use of low-dose CT protocols can reduce the radiation exposure with a comparable diagnostic accuracy with standard CT protocols [25]. These protocols could be a cost-efficient alternative to inpatient observation in patients with intermediate probability for AA. Nevertheless, not all emergency care units have 24-h availability of CT, and non-selective CT imaging is known to show lower sensitivity, mainly in patients with low or high clinical suspicion of AA [8, 26]. The results of this study indicate that most patients can be classified either in the high or the low probability group following the diagnostic algorithm, and thus, the need for diagnostic imaging can be significantly reduced. Moreover, the data of accuracy of abdominal ultrasound demonstrate that CT imaging is not required in all patients to detect the correct diagnosis. The diagnostic accuracy of ultrasound here was comparable with the data from literature with a sensitivity and specificity of 58% and 97%, respectively [26, 27]. The rather low sensitivity is likely explained by the fact that members of the surgical team, as it is standard of care in Germany, performed the ultrasound and not a certified ultrasound expert. With regard to these results, ultrasound and CT should be considered as complementary techniques. Ultrasound should be the preferred primary imaging modality and enables to rule in patients with risk for appendicitis. CT imaging is needed only in cases with equivocal clinical or ultrasound examination. This means less radiation exposure for the patient and cost saving for the hospital as currently shown in a meta-analysis from the USA [28].

The literature consistently reports at least twice the NAR in women than in men [4, 20, 29–32]. Only Antevil et al. [17] achieved an equally high NAR in women and men, but performing CT scan in every female patient. The primary aim of a new diagnostic pathway should therefore be to decrease the NAR in women of childbearing potential and protect them from unnecessary radiation exposure. In the current study, by the introduction of a standardized diagnostic workflow for patients with right-sided lower abdominal quadrant pain, a relevant reduction in NAR and, here especially in women (reduction of NAR from 16.5 to 4.4%; see Table 5) with minimal use of CT imaging was achieved. Only 20.8% of all women received a CT scan, and 18.5%, of men. This improvement can certainly also be explained by the routine gynecological consultation of all women of childbearing age as established in our diagnostic pathway. However, a routine gynecological examination is sometimes not practicable and may lead to considerable waiting times for the patient. As a result, also in our patient cohort, 37 patients were excluded due to a lack of consultation.

One of the major concerns about introducing a diagnostic pathway is an increased perforation rate due to a delay in treatment [33, 34]. At the same time, it has been shown that perforated appendicitis rates are not influenced by in-hospital delay and have not significantly changed with the increasing use of preoperative CT imaging [35, 36]. Andersson et al. [14] showed that reassessment after in-hospital observation and selective imaging in patients with an equivocal diagnosis is not associated with an increased risk for perforation and thus safe and efficient. The perforation rate in our study was 24.6%, similar to previously reported rates [19]. Neither the number of CT scans performed nor the time from admission to surgery significantly differed between patients with uncomplicated or perforated appendicitis. It is also noteworthy that the establishment of a clinical pathway accelerated the time from the first contact and interview to diagnosis in the DIALAPP study. As mentioned before, the time from admission to operation has been reduced in comparison with our previous institutional data [18]. This compares favorably with the results reported in the literature that the quality survey should focus on the accuracy of diagnosis rather than on its potential delay in therapeutic intervention.

A further aspect of quality improvement is the optimization of healthcare-related costs and resource utilization. Our study design does not allow a reliable statement on reduction of costs, but it allows an assessment of the potential savings. Thus, the reduction of NAR and CT scans by half at our institution has the potential for a saving of 6.7 million € per 100,000 suspicious cases for AA. Consistent implementation of the algorithm could even save up to 19.8 million € per 100,000 cases.

Similar previous studies have demonstrated that a minimization of negative appendectomies can provide financial and resource savings [37]. Scott et al. [38] showed that risk stratification and discharge of patients with low risk for appendicitis resulted in a significant reduction in admissions, preserving the availability of beds, diagnostic imaging, and financial resources.

Taken together, the simple diagnostic algorithm evaluated here showed high accuracy and the potential for an easy implementation into daily practice. The risk stratification based on objective scoring and a well-structured algorithm facilitated decision-making, particularly for the less experienced frontline surgeon who is often responsible for the initial assessment of patients. The pragmatic setup of our study represents a common clinical setting and thus increases generalizability of the study findings. We studied an unselected population of patients with acute right lower quadrant abdominal pain with a realistic sample prevalence of appendicitis. The application of the algorithm by all members of the surgical team reflects daily clinical applicability and allows scalability to other hospitals and settings.

There was no missed appendicitis in our patient cohort, taking into account the relatively high rate of lost to follow-up. Some patients who were discharged with a very low probability of having appendicitis and who were unresponsive to telephone calls may have been readmitted and operated at another hospital. Thus, we had no absolute confirmation of the absence of AA in the non-operated patients. This limitation is also a problem and has been reported as potential bias in other studies. A further limitation of this study is that it represents only a single center’s experience. Moreover, the number of exclusions was higher than expected, which increased the risk for statistical errors of the primary outcome measure “negative appendectomies.” This high rate of exclusion may also have led to a selection bias by the inclusion of more severe cases indicated by the high rate of perforated appendicitis and the exclusion of less severe cases indicated by a negative appendectomy rate of 15.6% in the group of excluded patients due to severe protocol violation. This high selection may have influenced the accuracy of the study.

Finally, our institution had not implemented antibiotic treatment for cases of imaging-confirmed uncomplicated appendicitis. This might have minimized the group of early appendicitis and unnecessary appendectomies which would otherwise be resolved by antimicrobial therapy alone. Nevertheless, antibiotic therapy alone for acute appendicitis shows a treatment effectiveness of only 72.6%, resulting in an appendectomy in 26.5% of patients initially treated conservatively as shown by the meta-analysis of Harnoss et al. [39]. Moreover, the incidences of complicated appendicitis and the hospital stay are significantly increased in the antibiotic treatment group in comparison with the surgical treatment group [39]. Thus, although antibiotics may prevent some patients from appendectomies, surgery still represents the gold standard of care for acute appendicitis.

The presented results support the implementation of a pathway in the diagnosis of AA. Patients with an uncertain clinical diagnosis and ultrasound result for appendicitis can safely be re-evaluated during a hospital admission. This risk-adjusted approach has the potential to reduce the number of unnecessary operation, simultaneously minimizing CT use.

## Conclusion

Implementation of a diagnostic algorithm combining clinical risk score, ultrasound, gynecological consultation, and selective CT imaging after re-evaluation resulted in high accuracy for the diagnosis of acute appendicitis. A low negative appendectomy rate can be achieved without increasing the risk for perforation from a delay in treatment.

## Data Availability

The datasets generated during the current study are not publicly available due to the terms agreed with the local ethics committee. They are available from the corresponding author on reasonable request.
